# To Evaluate the Impact of Ho:YAG Laser Lithotripsy for Ureteroscopic Removal of Proximal and Distal Ureter Calculi

**DOI:** 10.7759/cureus.47498

**Published:** 2023-10-22

**Authors:** Supran Sharma, Vilas Sabale, Vikram Satav, Abhirudra Mulay

**Affiliations:** 1 Urology, Dr. D. Y. Patil Medical College, Hospital & Research Centre, Pune, IND

**Keywords:** stone free, lithotripsy, holmium-yag laser, ureteroscopy, ureteral calculi

## Abstract

Background

Urinary calculus illness is a prevalent clinical issue encountered by the medical community, particularly urologists, in contemporary society. Laser technologies have been widely accepted as standard modalities for lithotripsy applications. Using the Ho:YAG laser has expanded the range of applications for ureteroscopic stone management (URS), enabling the treatment of bigger stones in all regions of the upper urinary tract. It is noteworthy that ureteroscopy (URS) demonstrates superior rates of stone clearance for distal stones, regardless of their size, with a success rate of 94.5% compared to 74% for other treatment modalities. Significant variation exists in the reported results and problems associated with Ho:YAG laser lithotripsy across different trials, as documented in the literature. The procedure's outcome might vary based on factors such as the size of the stone, the length of impaction, the presence of ureteral damage and granulation, the kind and size of endoscopes used, and the specific energy settings employed by various operators. The present study aimed to evaluate the impact of Ho:YAG laser lithotripsy for ureteroscopic removal of proximal and distal ureter calculi.

Methods

This prospective observational study was carried out in the Department of Urology at DY Patil Medical College and Hospital, Pune, from March 2021 to March 2023. Patients diagnosed with a case of ureteric stone who opted for URSL during the study period were included. A total of 50 patients who underwent URSL in the urology department were included in this study. These were then grouped into those with proximal ureteral stones and distal ureteral stones. (25 each)

Results

The study observed that patients diagnosed with proximal ureteral stones had bigger calculi, with a mean stone size of 15mm, in comparison to patients with distal ureteral stones, with a mean stone size of 10mm (P=0.010). The stone burden was significantly higher for proximal ureteral stone patients than those with distal ureteric stones (P=0.010). The average duration of the operating procedure for upper ureter stones was 70 minutes, but for lower stones, the mean operative time was 45 minutes (P<0.001). No statistical significance was seen in the median age of patients between the two groups (P=0.89). The maximum number of cases in the upper stone group were in the age group of 16-30 years, and in the lower stone group was in the age group of 31-45 years. The prevalence of DJ stents at the time of presentation was higher among patients diagnosed with proximal ureteric stones than those with distal ureteric stones, with rates of 28% and 20%, respectively (P=0.508). Full fragmentation was successfully accomplished in all patients within the distal calculus group, accounting for 100% of the cases. At the same time, for proximal ureteric stones, a single laser lithotripsy session resulted in 92% (23 patients) achieving a stone-free status after two weeks.

Conclusion

The study observed that stone size, burden, and procedure duration were statistically significant among other criteria. Mean age, stone HU, prior DJ stent, and stone-free rate were statistically insignificant. The procedure indicated that Ho:YAG laser lithotripsy has efficacy in treating both proximal and distal ureteral stones, with minimal intraoperative and postoperative complications. None of the complications were due to laser energy.

## Introduction

Urinary calculus illness is a prevalent clinical issue encountered by the medical community, particularly urologists, in contemporary society. Urinary stones have been a prevalent condition affecting humans for ages, with historical evidence reaching back to 4000 BC [1]. This ailment is well recognized as one of the most frequently occurring disorders inside the urinary system. The prevalence of ureteric calculi over an individual's lifespan is very elevated, with an estimated occurrence rate of roughly 12% among males and 7% among women. Most patients often exhibit symptoms between the age range of 30 to 60 years, with the highest occurrence seen between the ages of 35 and 45 years [2]. Recent research has shown a rise in the occurrence of urolithiasis during the last several decades. The increasing phenomenon is hypothesized to be linked to changes in lifestyle and eating patterns [3-5].

Laser technologies have been widely accepted as standard modalities for lithotripsy applications [6]. The use of the Ho:YAG laser has expanded the range of applications for ureteroscopic stone management (URS), enabling the treatment of bigger stones in all regions of the upper urinary tract [7]. In addition, advancements in the design of ureteroscopy and endoscopic tools have facilitated ureteroscopy (URS) as a less invasive alternative to open surgery for treating urinary calculi. This transition has occurred within the last decade. The guidelines on nephrolithiasis from the European Association of Urology (EAU) and the American Urological Association (AUA) have emphasized the modifications in the therapy of ureteral stones. It is noteworthy that ureteroscopy (URS) demonstrates superior rates of stone clearance for distal stones, regardless of their size, with a success rate of 94.5% compared to 74% for other treatment modalities [8].

Research findings indicate that the efficacy of ureteral stone removal (URS) is influenced by several factors, including the dimensions and location of the stone, the quantity, and composition of the stone fragments, as well as the specific kind of lithotripter device used [9,10]. With the progression of technology, several energy sources, including pneumatic, electrohydraulic, laser lithotripters, and ultrasonic methods, have been used for stone fragmentation [11,12]. 

Significant variation exists in the reported results and problems associated with Ho:YAG laser lithotripsy across different trials, as documented in the literature. The procedure's outcome might vary based on factors such as the size of the stone, the length of impaction, the presence of ureteral damage and granulation, the kind and size of endoscopes used, and the specific energy settings employed by various operators.

The present study aimed to evaluate the impact of Ho:YAG laser lithotripsy for ureteroscopic removal of proximal and distal ureter calculi.

## Materials and methods

This was a prospective observational study carried out in the Department of Urology at DY Patil Medical College and Hospital, Pune, from March 2021 to March 2023 after obtaining clearance from the Institutional Ethics Sub-Committee (Research Protocol No.IESC/S.SP/2020/06). Patients diagnosed with a case of ureteric stone who opted for URSL were included in the study. A total of 50 patients who underwent URSL in the urology department were included and grouped into two groups based on diagnosis and location proximal ureteral stones and distal ureteral stones (25 each). 

The research comprised individuals with symptomatic ureteric stones who were assessed for eligibility after informed consent. Patients having bleeding diathesis, pregnancy, current UTI, leucocytosis > 11000/cumm, acute renal failure, CKD patients with GFR < 20ml/h, skeletal abnormalities, congenital renal abnormalities, positive urine culture, and patients not giving consent were excluded from the study. All participants underwent a comprehensive assessment, including medical history collection and clinical examination. Additionally, various laboratory tests were conducted, such as complete blood count, routine and microscopic urine analysis, urine culture, liver function test, renal function test, coagulation profile, serum sodium, and potassium levels, as well as screening for Hepatitis B, Hepatitis C, and HIV. Furthermore, renal ultrasound, KUB (kidneys, ureters, and bladder) imaging, and CT urography were performed. The surgical indications were established, and the patient had the procedure after confirmation of their eligibility for anesthesia. The URSL procedure was conducted using the usual approach as described below.

The surgery was conducted under spinal anesthesia. To mitigate bias, all surgeries were conducted by a single surgeon. The duration of the operative procedure was determined by measuring the time from the initiation of anesthesia to the completion of instrument removal. The operation commenced with the use of cystoscopy. A guidewire with a diameter of 0.035 inches was inserted into the ureter, and its placement was verified using fluoroscopy. An 8-French newborn feeding tube was inserted into the bladder for drainage.

The procedure of ureteroscopy was conducted using a Storz semi-rigid 7 Fr ureteroscope. The object's length was 43 centimeters, and it had a working channel of 3.2 French units, including a rounded and atraumatic distal tip. The process of stone fragmentation was conducted using the Quanta System 60W Ho:YAG laser in conjunction with a 365 μm fiber. The energy range in the experiment was between 0.6 Joules and 1.4 Joules, while the frequency range was measured between 5 Hz and 15 Hz. The laser dusting method was used to mitigate the phenomenon of stone retropulsion. Following the established procedure of our institution, DJ stents were inserted in all instances. The measurement of stone size was determined by the maximal diameter of the stone as seen using ultrasound KUB/ CT urography. The term "stone burden" refers to the calculated value obtained by determining the maximal diameter of a stone in two dimensions using either Kidney Ureter Bladder (KUB) ultrasonography or CT urography.

After the first intervention, patients were monitored on an outpatient basis after two weeks, using abdominal ultrasonography and X-ray KUB to determine the rate of stone clearance. The evaluation of the procedural outcomes included the stone-free rates and the occurrence of complications. The stone-free rate refers to the proportion of instances where all calculi are pulverized into pieces or fine dust measuring 2 mm in diameter or less, as seen using abdominal ultrasonography or x-ray imaging two weeks after the surgery. The occurrence of bleeding was regarded as a complication when its severity necessitated the discontinuation of the procedure or the administration of a blood transfusion. All post-operative complications, including fever, mucosal damage, ureteric avulsion, transitory renal dysfunction, sepsis, other organ injury, and mortality from the surgery, were observed and classified according to the Modified Clavien Dindo Grading system.

Statistical analysis

The data analysis was entered in EXCEL and analyzed using Jamovi version 2.3.28. The categorical data was represented using numerical values and expressed as percentages. The mean ± standard error was used to represent the continuous variables if normally distributed and Median(IQR) if not normally distributed. The normality of the data distribution was assessed by the Shapiro-Wilk test. Parametric tests, i.e., t-test, were used for data that followed a normal distribution, with the Welch modification if homogeneity of variances was absent. Non-parametric tests, i.e., the Mann-Whitney test, were utilized for data that did not adhere to a normal distribution. The Chi-square test/Fisher's exact test was used to examine qualitative variables. A P value of <0.05 was considered statistically significant.

## Results

Table 1 shows no significant difference in the median patient's age between the two groups. The male-to-female ratio was higher in the proximal group.

**Table 1:** Mean age and gender distribution. t-test, Chi-square test

	Proximal Stone (25)	Distal stone (25)	p-value
Age (years)	43.6±15.8	44.2±17.6	0.89
Gender (Male/Female)	18/7	14/11	0.54

Table 2 shows that the youngest patient in our study group was a six-year-old girl. The maximum number of cases in the upper stone group was in the age group of 16-30 years; in the lower stone group, the maximum cases were in the age group of 31-45 years.

**Table 2:** Age distribution

Age in years	Proximal Ureter Stones		Distal Ureter Stones	
	Number of cases	Percentage	Number of cases	Percentage
<15	0	0	1	4%
16-30	8	32%	4	16%
31-45	6	24%	11	44%
46-60	6	24%	3	12%
>60	5	20%	6	24%

Table 3 shows that patients with proximal ureteral stones had significantly larger calculi (mean stone size = 15 mm) vs. those with distal ones (mean size= 10 mm).

**Table 3:** Average stone size. Mann-Whitney Test

	Proximal ureteric calculi (mm)	Distal ureteric calculi (mm)	p-value
Average stone size (Median IQR)	15(10-15)	10(8-12)	0.010

Table 4 shows that the stone burden for proximal ureteral stone patients was significantly more than that of patients with distal ureteric stones.

**Table 4:** Mean stone burden. Mann-Whitney Test

	Proximal ureteric calculi (mm^2^)	Distal ureteric calculi (mm^2^)	p-value
Mean Stone Burden (Median IQR)	150(80-150)	85(64-113)	0.010

Table 5 shows that the average Hounsfield unit for stones on computed tomography was almost similar for both groups.

**Table 5:** Average Hounsfield Unit. t-test

	Proximal ureteric calculi	Distal ureteric calculi	p- value
Average HU	1096±158	1049±159	0.299

Table 6 shows that patients with proximal ureteric stones had more DJ stents at presentation than those with distal ureteric stones (28% vs 20%). A few of the reasons cited are obstructed uropathy and sepsis. However, the difference was not statistically significant.

**Table 6:** Prior DJ Stent. Chi-square test

Prior DJ Stent	Proximal Ureteric Calculi	Distal Ureteric Calculi	p- value
YES	7 (28%)	5 (20%)	0.508
NO	18 (72%)	20 (80%)	

Table 7 shows a significant difference in operative time (from induction to removing all instruments from the patient) for proximal and distal ureteric stones. The mean operative time for upper ureteric stones was 70 minutes, while for lower ureteric stones, it was 45 minutes.

**Table 7:** Mean Operative Time Welch Test

	Proximal Ureteric Calculi	Distal Ureteric Calculi	P Value
Mean Operative Time (in minutes)	69.6±22.0	44.6±13.8	<0.001

Table 8 shows that complete fragmentation was achieved in all patients of the distal calculus group (100%), while in those with proximal ureteric stones, 23 patients (92%) were rendered stone-free after two weeks by a single laser lithotripsy procedure. The difference in stone-free rate was found insignificant.

**Table 8:** Stone Free Rates Fisher's exact test

	Proximal Ureteric Calculi	Distal Ureteric Calculi	p- value
Stone Free Rates (2 weeks)	92%	100%	0.49

As shown in (Table 9), perioperative complications were recorded in three patients with proximal ureteric stones, while none was encountered in the distal ureteral stone group. The ureteral mucosal injury was seen in one patient, and two patients had retropulsion of stone. Post-operativePostoperative complications were seen in four patients in each group. None of the complications were due to laser energy. Overall complication rate was 18.33%.

**Table 9:** Peri-and Post-Operative Complications.

Complications	Upper Ureteric Stone (25)	Lower Ureteric Stone (25)	p-value
Retropulsion	8% (2/25)	0 %	0.14
Injury to Ureter	4% (1/25)	0 %	0.317
Post-operative Infection	16% (4/25)	16 % (4/25)	1
Blood Transfusion	0 %	0 %	
Urinary Extravasation/ Urinoma	0 %	0 %	

As shown in (Table 10) intraoperative complications were classified as Grade Illa (small mucosal laceration without leakage and retropulsion) and post-operative complications as Grade II (febrile urinary tract infection) according to the modified Clavien Dindo grading system.

**Table 10:** Modified Clavien Dindo Classification of Peri- and Post Operative Complications.

Grade	Proximal Ureteral Stone N (%)	Distal Ureteral Stone N (%)
I	0	0
II	4 (16%)	4 (16%)
Illa	3 (12%)	0
Illb	0	0
IV	0	0
V	0	0

## Discussion

The widespread popularity of the Ho:YAG laser may be attributed to its excellent efficacy in fragmenting stones of various compositions. This crucial quality has been a limiting factor in adopting other laser types for lithotripsy. In addition to its substantial margin of safety and notable patient comfort rate, this particular intervention is considered an optimal selection for the therapy of ureteral stones. Furthermore, the field of urology has seen significant progress in instrument technology, including the development of the semi-rigid ureteroscope, breakthroughs in the next-generation flexible ureteroscope, improvements in irrigation systems, and enhancements in optical systems. These technological innovations have not only facilitated the work of urologists but have also been well-received by patients due to their enhanced longevity. According to the European Association of Urology (EAU) in 2020, there is a similar stone-free rate after ureteroscopy (URS) or shock wave lithotripsy (SWL) for the treatment of ureteral stones. Nevertheless, it has been shown that bigger stones tend to acquire a stone-free condition quicker when treated using ureteroscopy (URS). While ureteroscopy (URS) has shown efficacy in treating ureteral calculi, it is associated with a higher likelihood of complications [13].

In the present age of endourology, there has been a significant reduction in complications and morbidity related to ureteroscopy (URS) [14]. The European Panel conducted a comprehensive evaluation to evaluate the advantages and drawbacks of ureteroscopy (URS) in comparison to extracorporeal shock wave lithotripsy (ESWL) [15]. The findings indicate that compared to extracorporeal shock wave lithotripsy (ESWL), ureteroscopy (URS) is linked to a notably higher rate of successful elimination of kidney stones. Ureteroscopic lithotripsy exhibits a reduced need for retreatments and subsequent operations, yet it is associated with elevated complication rates and extended hospitalization periods. According to the European Association of Urology (EAU), ureteroscopy (URS) is more likely to achieve a stone-free status when performed as a single operation [13]. The findings of our research indicate that the rate of stone clearance for stones located in the proximal ureter after a single surgery was 92%, but it was 100% for distal ureteral calculi. Although there was a disparity in the stone-free rate between the two groups, the observed difference was not statistically significant (p-value = 0.49). This finding aligns with other literature publications showing the effectiveness of ureteroscopic Ho:YAG laser treatment for both proximal and distal ureteral stones. According to Khoder et al. (2014), the research revealed that the stone-free rate for lower ureteric stones was much higher at 100% compared to upper ureteric stones, which had a stone-free rate of 82.4% [16].

In the present investigation, it was observed that patients with proximal ureteral stones exhibited larger stone sizes, greater stone load, and longer operating times compared to patients with lower ureteric stones. However, it is noteworthy that these parameters mentioned above did not significantly impact the stone-free rate. The results of many authors in prior investigations showed divergence. In their study, Khoder et al. (2016) observed a correlation between the stone load and size (diameter) of the biggest stones and the success rate of ureteroscopy with Holmium: YAG laser lithotripsy, specifically in terms of achieving a stone-free state [16]. In contrast to the findings of Purpurowicz et al. (2012), who reported stone-free rates of 85.2% for upper ureteric calculi and 90.0% for lower ureteric calculi using Ho:YAG laser ureteroscopic lithotripsy (URSL). A total of 54.9% of patients had stenting after ureterorenoscopy with laser lithotripsy (URSL), as reported in a study including 53 participants [17]. Our study exhibited similarities to the abovementioned research, particularly regarding the observed clearance in the distal ureter. However, it is worth noting that our study achieved a notably high rate of clearance. All participants in our research had stent placement after the operation. In research conducted by Jiang H et al. in 2007, it was determined that the stone-free rate for distal ureteric calculi was 100%, whereas for proximal ureteric calculi, it was 70.3%. Incomplete fragmentation occurred during laser lithotripsy in 49 patients, accounting for 7.03% of the cases, mostly due to retrograde stone migration. The incidence of intraoperative complications was found to be 1.15%. These complications included ureteral perforation caused by the ureteroscope in two cases, the guidewire in three cases, and the laser in three cases. Ureteral stenting was used to control little damage effectively [18].

In our hospital, it is customary to do post-operative stenting for three weeks after ureterorenoscopic lithotripsy (URSL) in all patients who have presented with large and/or impacted calculi, regardless of the location, presence of ureteral mucosal damage, single kidney, or retropulsion. At our facility, most procedures are performed by resident trainees under the guidance and oversight of the faculty members. This finding elucidates the rationale for our elevated rates of post-operative stenting, which stand at 100% for both proximal and distal stones. In a patient presenting with a proximal impacted stone with ureteral mucosal damage, a stent with an extended lifespan of 4 weeks was inserted. Ureteroscopic laser lithotripsy with the holmium laser has shown a high level of safety, particularly in cases where extracorporeal shock wave lithotripsy (ESWL) is anticipated to be ineffective or contraindicated. Most problems documented in prior research mostly consisted of mild intra- and post-operative issues. According to Sofer et al. (2002), the incidence of problems associated with Ho:YAG laser treatment is as low as 1% [19]. No significant problems were seen in our investigation. The overall rate of complications was 18.33%. Three patients with proximal ureteric stones were found to have perioperative problems, but no issues were seen in the distal ureteral stone group. The observed cases were categorized as Grade Illa, characterized by minimal mucosal damage without extravasation, including two instances exhibiting retropulsion. The findings of our study provide a notable discrepancy when compared to the research conducted by Manohar et al. (2008), which revealed a retropulsion rate of up to 24% with Ho:YAG lithotripsy for upper ureteral stones [20], while our data demonstrate a contrasting outcome. The poor retropulsion rate observed in the present investigation may be ascribed to the use of low-energy settings for proximal calculi in our experimental setup. Eight patients had post-operative problems, including four individuals in each category having ureteric stones. The cases were categorized as Grade II, specifically febrile urinary tract infection, following the modified Clavien Dindo grading system. All of these individuals showed positive responses to parenteral administration of antibiotics. The primary limitation of this research is its small sample size since it only included 50 patients. Therefore, it is essential to conduct research including a larger cohort of patients in both groups to verify these findings.

## Conclusions

The stone-free rate showed no significant difference between proximal and distal ureteric stones when using ureteroscopic Ho: YAG laser lithotripsy, with 92% and 100% rates, respectively. The procedure indicated above results in a significant increase in total stone freedom. The Ho: YAG laser lithotripsy is a suitable instrument with a minimal incidence of complications for treating both proximal and distal ureteral stones. Both intraoperative and post-operative problems are mostly minor, often requiring only cautious care.
